# Commentary: Conservative Management of a Scar Abscess Formed in a Cesarean-Induced Isthmocele

**DOI:** 10.3389/fsurg.2017.00049

**Published:** 2017-09-06

**Authors:** Ekaterini Christina Tampaki, Athanasios Tampakis, Konstantinos Kontzoglou, Gregory Kouraklis

**Affiliations:** ^1^Second Department of Propaedeutic Surgery, Laiko General Hospital, School of Medicine, National and Kapodistrian University of Athens, Athens, Greece; ^2^Department of Visceral Surgery, Basel University Hospital, Basel, Switzerland

**Keywords:** scar abscess, uterine discharge, conservative treatment, antibiotic choice, cesarean-induced isthmocele

We read with great interest the article by Boukrid et al. on the very interesting case of a primiparous woman presenting a symptomatic abscess in the isthmocele 10 years after a cesarean section (CS) (1). Any post-caesarian wound infection such as a scar abscess is caused usually due to a bacterial infection in the surgical incision site, commonly developing with signs of lower abdominal pain, fever, and wound sensitivity. In fact, many CS wound infections usually appear within the first couple of weeks after delivery and are diagnosed at follow-up visits. What makes, however, this case so interesting is the development of the surgical site infection (SSI) 10 years after the CS took place.

First, we certainly agree that the presentation of this so rare case brings up excellent discussion topics on the subject. Therefore, interesting to know regarding this patient would be if there were specific risk factors for any post-SC wound infection occurrence, including obesity, immunosuppression, or existence of diabetes in the patient’s history. Moreover, presence of chorioamnionitis during labor, poor prenatal care (not so significant in this case because of the 10-year occurrence of the ISS), previous C-sections that were indeed denied by the patient, not administering prophylactic antibiotics or pre-incision antibacterial care, a prolonged lasting labor or switch to surgery, and extreme blood loss during the procedure seem also crucial. Interestingly, many SSIs have been described in patients who smoke as well as after use of long-term systemic corticosteroids. According to Chunder et al. in a randomized controlled prospective study in Africa, they positively correlated the nylon or staple sutures use after a CS with higher infection risk. However, polyglycolide sutures are favored because of their absorbability and biodegradability (2), whereas skin closure with subcutaneous contrarily to staple sutures minimizes the superficial infections rates (3).

Apart from that, of high significance appears the categorization of the types and the appearance of the related infections after a CS, being usually classified as wound-related cellulitis or abdominal abscess, contributing to the most common post-SC infections including endometriitis and pelvic cellulitis (4). As CS wound infections may also spread and even cause serious complications such as necrotizing fasciitis, fascia rupture, or even wound dehiscence and in worst cases evisceration with septicemia or even progression to septic shock, the correct antibiotic treatment is highly important to clear up the infection, with common choices including β-lactam antibiotica administration as they specifically need to target staphylococcal and streptococcal bacteria. Interestingly, patients should be treated along with antibiotics they receive ahead for pelvic cellulitis or endometriitis therapy, whereas 1 g of vancomycin every 12 h could also be administered due to the subsequent increase in MRSA resistance (4).

Along with the worst complications being presented so far in patients undergoing major procedures such as CS, the septic pelvic vein thrombophlebitis occurs in approximately 0.5–1% of these patients (5). Usually, some of these patients present with a palpable mass caused by a thrombus appearing most often in the right ovarian vein or alternatively patients present with multiple small thrombi in the pelvic vasculature. The treatment being offered apart from the broad-spectrum intravenous antibiotics appears as a good regimen covering adequately the treatment of severe, polymicrobial pelvic infections and includes clindamycin or metronidazole plus penicillin or ampicillin plus gentamycin. Additionally, the patients receive for 7–10 days an unfractionated heparin or low-weight heparin (5).

Interestingly, Sullivan et al. proved in a randomized controlled trial that women who received antibiotic prophylaxis 15–60 min before the start of surgery in comparison to those who received prophylaxis after clamping of the umbilical cord, presented significantly lower rates of endometriitis (6), whereas cefazolin (1 g intravenous or intramuscular) has also been described as an excellent choice for prophylaxis.

However, the patient presented has been fruitfully conservatively managed with a two-weekly administration of amoxicillin–clavulanic acid 1 g/12 h, with successful and complete resolution of the abscess. However, we would like to highlight the importance of proper treatment selection along with careful consideration of the patient individual characteristics and thorough history related to treatment will, especially in such rare cases presented, that could possibly lead us in the future to decreased complication rates, readmissions, number of postoperative visits, and a better objective understanding of the cause. Anaphylactic reactions have been recorded as adverse effects and correlated with penicillins, especially the injectable forms, further damaging the patients. Furthermore, the lack of randomized trials underlines the lack of widespread implementation of screening guidelines regarding the best option when treating conservatively such patients. In practice, necessity to prioritize by first identifying post-SC infections high-risk situations, and administering prophylactic antibiotics as soon as possible by simultaneously minimizing the risk of an anaphylactic reaction by choosing the antibiotic based on maternal allergy history or avoiding it whatever the cause, seems mandatory.

Concluding, after investigation of all the abovementioned risk factors combined with an efficient choice of antibiotic prophylaxis prior and after cesarean delivery, is by all means the best technique approaching the subject when opting for conservative versus surgical patient management. Therefore, we could minimize the potential risk of SSI development in a short- and in long-term period of time maybe, even extremely rare, also after 10 years after the CS has taken place.

## Author Contributions

ECT conceived of the idea and wrote the manuscript. AT helped draft the manuscript. KK helped to revise the manuscript. GK helped to revise the manuscript. All the authors read and approved the final manuscript.

## Conflict of Interest Statement

The authors declare that the research was conducted in the absence of any commercial or financial relationships that could be construed as a potential conflict of interest. The reviewer, KB, and handling editor declared their shared affiliation.

## Abbreviations

CS, cesarean section; SSI, surgical site infection; PGA, polyglycolide.
